# Patterns of Genetic Variation Reveal Plant's Evolutionary Roots

**DOI:** 10.1371/journal.pbio.0030231

**Published:** 2005-05-24

Biologists have developed ever more sophisticated ways to find molecular traces of natural selection. These traces—which occur as variations in DNA sequence, or polymorphisms, between and within species—are thought to harbor the genetic basis of adaptive events.

The study of natural selection at the molecular level has long been dominated by Kimura's theory of neutral evolution, which argues that most polymorphisms (in both DNA and protein sequence) have minor or no selective effect, and are governed by random, not selective, processes. The strength of this theory is that it leads to clear predictions that can be tested to identify those polymorphisms that really are subject to selection. Even though there's a large body of literature devoted to the statistical testing of selective neutrality, these tests are generally based on theoretical models, the assumptions of which have largely been untested. Only recently have the necessary quantities of data for testing the merits of these models become available, thanks to high-throughput genotyping and sequencing technologies.

Working with Arabidopsis thaliana, the first plant genome sequenced, Magnus Nordborg, Joy Bergelson, and their colleagues investigate a global survey of polymorphism patterns in the genomes of 96 plants. The scale of their study affords robust insights into the genomic pattern of polymorphism of the plant and sheds light on its demographic history. The results also lay the foundation for future work on the genetic basis of A. thaliana variation while challenging the assumptions of standard mathematical models for determining whether a gene is under natural selection in the plant.

Nordborg and colleagues sequenced 876 short genome fragments of 96 A. thaliana plants from both worldwide natural populations and laboratory stocks. In total, they described 44,000,000 DNA bases of genetic material, which revealed 17,000 polymorphisms, either in the form of single changes in DNA sequence (single nucleotide polymorphisms, or SNPs) or as losses or additions of DNA sequence between individual plants.

The level of polymorphism in A. thaliana is unexpectedly high for a plant that is highly self-fertilizing. To see if these polymorphisms were uniformly shared across plant populations, or had a distinct structure, Nordborg and colleagues grouped a subset of the A. thaliana plants into populations based on their geographic origin. Although they found that individuals within a population harbor much of the variation that is typical of the species worldwide, it appeared that some of the variation was specific to particular geographic regions. Furthermore, closely related plants were almost always from the same local region. The authors suggest that is what would be expected for a sexually reproducing species found worldwide. [Fig pbio-0030231-g001]

**Figure pbio-0030231-g001:**
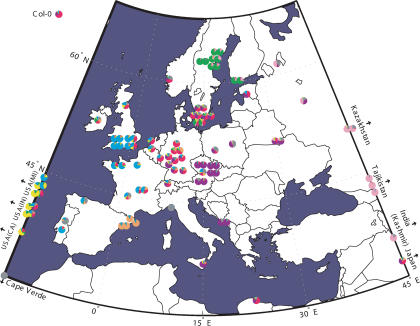
The global geographic structure of variation in Arabidopsis is shown by the clusters of similar pie charts signifying patterns of isolation (each chart represents an Arabidopsis accession)

With such a large data set, it also becomes possible to see if the underlying assumptions of mathematical models commonly used for determining whether a gene is under selection are appropriate for A. thaliana. Nordborg and colleagues found that the patterns they observe do not fit the standard neutral model of evolution, which is expected to explain most genetic variation. This model is the benchmark against which researchers pinpoint the signature of selection at particular genes. The authors caution that “commonly used ‘tests of selection’ are simply not valid in A. thaliana.”

Nordborg and colleagues have provided a wealth of detail to our understanding of genetic variation in Arabidopsis on a genome-wide scale. Future research can now begin to use this Arabidopsis genetic footprint to find the exact variations that contribute to useful plant traits—and plumb its genome for evolutionary clues.

